# iTRAQ-Based Proteomic Analysis reveals possible target-related proteins and signal networks in human osteoblasts overexpressing FGFR2

**DOI:** 10.1186/s12953-018-0140-x

**Published:** 2018-06-19

**Authors:** Tianyi Cai, Baojin Wu, Xinjie Tang, Zhaoping Zhou, Junyi Yang, Ronghu Ke, Xiongzheng Mu

**Affiliations:** Department of Plastic and Reconstructive Surgery, Huashan Hospital, Fudan University School of Medicine, No. 12, Wu Lu Mu Qi Road (M), Shanghai, 200040 China

**Keywords:** iTRAQ, FGFR2 osteoblast

## Abstract

**Background:**

Fibroblast growth factor receptor 2 (FGFR2) play a vital role in skeletogenesis. However, the molecular mechanisms triggered by FGFR2 in osteoblasts are still not fully understood. In this study, proteomics and bioinformatics analysis were performed to investigate changes in the protein profiles regulated by FGFR2, with the goal of characterizing the molecular mechanisms of FGFR2 function in osteoblasts.

**Methods:**

In this study, FGFR2-overexpression cell line was established using the lentivirus-packaging vector in human osteoblasts (hFOB1.19). Next, the isobaric tags for relative and absolute quantitation (iTRAQ) in combination with the liquid chromatography-tandem mass spectrometry (LC-MS/MS) method was used to compare the proteomic changes between control and FGFR2-overexpression cells. Thresholds (fold-change of ≥ 1.5 and a *P*-value of < 0.05) were selected to determine differentially expressed proteins (DEPs). The bioinformatics analysis including GO and pathway analysis were done to identify the key pathways underlying the molecular mechanism.

**Results:**

A Total of 149 DEPs was identified. The DEPs mainly located within organelles and involved in protein binding and extracellular regulation of signal transduction. ColI, TNC, FN1 and CDKN1A were strikingly downregulated while UBE2E3, ADNP2 and HSP70 were significantly upregulated in FGFR2-overexpression cells. KEEG analysis suggested the key pathways included cell death, PI3K-Akt signaling, focal adhesion and cell cycle.

**Conclusions:**

To our knowledge, this is the first protomic research to investigate alterations in protein levels and affected pathways in FGFR2-overexpression osteoblasts. Thus, this study not only provides a comprehensive dataset on overall protein changes regulated by FGFR2, but also shed light on its potential molecular mechanism in human osteoblasts.

**Electronic supplementary material:**

The online version of this article (10.1186/s12953-018-0140-x) contains supplementary material, which is available to authorized users.

## Background

A complex network that regulates the differentiation of mesenchymal stromal cells into osteoblasts and terminal differentiation into osteocytes under appropriate stimulation controls bone development. Although the development occurs via two types of processes known as intramembranous ossification and endochondral ossification, osteoblasts are its major bone forming cells for bone deposition in both processes [1]. The functions of bone-producing osteoblasts are significantly regulated by transcription factors in a spatially and temporally controlled manner [2]. Thus, study of the factors that regulate osteoblasts takes insight into the key to the bone development.

Fibroblast growth factor receptor 2 (FGFR2), a member of the FGF receptor family, controls cell growth, differentiation and survival in multiply tissues [3]. Among these tissues, the skeleton is an important target for FGFR2, which is expressed in condensed mesenchyme and later in sites of endochondral and intramembranous ossification. Notably, FGFR2 is involved in osteoblastic differentiation and survival [4, 5]. Consistent with an important role of FGFR2 signaling in the control of osteoblast, FGFR2-deficient mice die of failure in limb buds [6]. More importantly, patients with FGFR2 mutation exhibit several types of syndromic craniosynostosis in an autosomal dominant manner, including Apert (MIM #101200) and Crouzon (MIM #123500) syndromes. These observations suggested FGFR2 is essential for the normal proliferation of osteoblasts and osteogenic gene expression during postnatal bone development. However, the molecular mechanisms underlying FGFR2-triggered osteoblastic functions remain fully unknown.

Based on this background, we subsequently established stable osteoblasts overexpressing FGFR2 mediated by lentivirus and systematically analyzed altered signaling triggered by FGFR2 in human osteoblasts. Finally, we demonstrated intracellular signaling pathways in osteoblasts with FGFR2 overexpression are indicative of a less proliferative profile as compared with profiles from control.

## Methods

### Cell Culture

The human fetal osteoblastic 1.19 cell line (hFOB) was obtained from American Type Culture Collection (ATCC, Manassas, VA) and were cultured as previously described [7, 8].

### Lentiviral transduction and establishment of cell line

The full length FGFR2 (NM_000141) was synthesized from human cDNA library using the following primers: forward primer 5’- CCAACTTTGTGCCAACCGGTCGCCACCATGGTCAGCTGGGGTCGTTTCATC-3’, reverse primer 5’- AATGCCAACTCTGAGCTTTGTTTTAACACTGCCGTTTATG -3’. The sequences were cloned into lentiviral vector GV341 (GeneChem, Shanghai, China) with IRES-GFP. Lentivirus particles were produced by co-transfecting 293T cells with lentivirus vectors and pHelper 1.0, pHelper 2.0 packaging plasmids (GeneChem, Shanghai, China). Supernatants containing virus particles were collected, filtered and concentrated. The hFOB cells were transduced the FGFR2 or vector virus particles to generate the FGFR2 and control (NC) sublines. To generate cells stably expressing the vector control or FGFR2 constructs, the transfected cell were selected with 1 μg/ml puromycin (Invitrogen,CA) and pools of selected cells were subjected to proteomic experiments [9]. Efficiency of GFP expression was analyzed by fluorescence microscope. FGFR2 expression was confirmed by real-time quantitative PCR and immunoblot analysis, respectively.

### RNA extraction and real time PCR

Total RNA was isolated from osteoblasts hFOB with the Trizol reagent (Invitrogen,CA) according to the manufacturer’s protocol. cDNA was synthetized using a PrimeScript RT reagent kit (Takara, Dalian, China). Target gene and endogenous control β-actin were amplified by qPCR using the SYBR Green PCR Kit (Takara, Dalian, China). Real-time PCR was performed in the ABI-Prism 7300 sequence detection system (Applied Biosystems, CA). The primers for PCR were as follows: FGFR2, forward, 5’- CCAACTGCACCAACGAACTG-3’, FGFR2, reverse 5’- ACTGTTCGAGAGGTTGGCTG-3’; Troponin I3 (TNNI3) primer: forward 5’-CGTGTGGACAAGGTGGATGAAG-3’, reverse5’-GCCGCTTAAACTTGCCTCGAAG-3’. Ubiquitin-conjugating enzyme E2 E3 (UBE2E3) primer: forward 5’GACAACTGGAGTCCCGCTTTGA-3’; reverse primer 5’-CTGAGTGGCTATGCTTCCAACC-3’; β-actin primer, forward, 5’- TTGTTACAGGAAGTCCCTTGCC-3’, β-actin, reverse, 5’- ATGCTATCACCTCCCCTGTGTG -3’. An initial activation step of 95°C for 15 s was followed by 40 cycles of 95°C for 5 s and 60°C for 30 s. The target gene expression was analyzed by 2^-**△△**Ct^ approach.

### Western blot

Osteoblasts were harvested on ice in phosphate-buffered saline (PBS) and centrifuged. To prepare cell protein, 10^6^ cells were lysed during 15 min on ice in 100 μl of lysis buffer (150mM NaCl, 1% Nonidet P-40, 0.1% SDS, 50mM Tris-HCl pH 8.0) containing protease inhibitor (1:100; Complete™ , Roche) and sodium orthovanadate (1 mM). Twenty μg proteins were separated by 10% SDS-PAGE and transferred to PVDF membranes (Millipore, Bedford, MA). Membranes were blocked for 1 hour in Tris-buffered saline-Tween (TBST; 20 mM Tris–HCl [pH 7.6], 137 mM NaCl, 0.1% Tween-20) containing 5% milk. The membranes incubated with anti-FGFR2 (1:1000, Abcam) or β-actin (1:5000, Sigma-Aldrich) antibodies at 4 °C overnight. Bound antibodies were detected by horseradish peroxidase (HRP)-conjugated rabbit anti-mouse antibody. The enhanced chemiluminescence (ECL) method was used for immunodetection and densitometry was performed using image J.

### Protein digestion and iTRAQ labeling

Protein digestion and iTRAQ labeling was performed as previously described by Yi Zhu et al [10]. For protein digestion, 200 μg of proteins for each sample were incorporated into 30 μL standard buffer (4% SDS, 100 mM DTT, 150 mM Tris-HCl pH 8.0). The detergent, DTT, and other low-molecular-weight components were removed using Buffer Y (8 M Urea, 150 mM Tris-HCl pH 8.0) by repeated ultrafiltration (Microconunits, 30 kD). Next, 100 μL 0.05 M iodoacetamide in UA Buffer was added to block reduced cysteine residues and the samples were incubated for 20 min in darkness. The filters were washed with UA Buffer three times and then twice with DS Buffer (50 mM triethylammonium bicarbonate at pH 8.5). Finally, the protein suspensions were digested with 2 μg trypsin (Promega) in 40 μL DS Buffer overnight at 37°C and the resulting peptides were collected as a filtrate. The peptide content was estimated by UV light spectral density at 280 nm. The peptide was labeled using the iTRAQ reagent according to the manufacturer’s instructions (Applied Biosystems) as follow: FGFR2-overexpressing osteoblasts (FGFR2: 114 tag, 115 tag), control osteoblasts (NC: 118 tag, 119 tag). The labelled peptide was were then pooled and vacuum dried.

### SCX fractionation

The iTRAQ-labeled peptide mixtures were fractionated by SCX chromatography using the AKTA Purifier system (GE Healthcare). The dried peptide mixture was reconstituted with 4 mL Buffer A (10 mM KH2PO4 in 25% of ACN, pH 2.7) and loaded onto a 4.6×250 mm Ultremex SCX column (Phenomenex, Torrance, CA). The peptides were eluted at a flow rate of 1 mL/min with a gradient of 0%–10% Buffer B (500 mM KCl, 10mM KH2PO4 in 25% of ACN, pH 2.7) for 10 min, 10–20% Buffer B for 10 min, 20%–45% Buffer B for 5 min, and 45%–100% Buffer B for 5 min. The UV absorbance at 214 nm was monitored when the fractions were collected. The collected fractions (about 36 fractions) were finally combined into 15 pools and desalted on C18 Cartridges [Empore SPE Cartridges C18 (standard density), 7 mm inner diameter, 3 mL volumes, Sigma]. Each fraction was concentrated by vacuum centrifugation and reconstituted in 40 μL of 0.1% (v/v) trifluoroacetic acid for liquid chromatography-tandem mass spectrometry (LC-MS/MS) [11].

### LC-MS/MS analysis and data screening

The LC fractions were analyzed using a Q Exactive MS (Thermo Finnigan) equipped with Easy nLC(Proxeon Biosystems, now Thermo Fisher Scientific). A 10 mL aliquot of each fraction was injected for nanoLC-MS/MS analysis. The peptide mixture was loaded onto the C18-reversed phase column (Thermo Scientific Easy Column, 10-cm long, 75-μm inner diameter, 3-μm resin) in Buffer A (0.1% formic acid) and separated with a linear gradient of Buffer B (80% acetonitrile and 0.1% formic acid) at a flow rate of 250 nL/min controlled by IntelliFlow technology over 60 min. The gradient included 0 to 40% (v/v) for 55 min, 40% to 100% (v/v) for 58 min, and 100% (v/v) for 60 min. MS data acquisition was performed using the 10 most abundant precursor ions from the survey scan (300–1800 m/z) for high-energy collisional dissociation (HCD) fragmentation. The target value for Automatic Gain Control (AGC) was 3e6. Dynamic exclusion for selected precursor ions was 60 s. The resolution was set as follows: 70,000 atm/z 200 for MS scan and 17,500 at m/z 200 for HCD spectra. The normalized collision energy was 30 eV and the under fill ratio was defined as 0.1%.

MS/MS spectra were searched using MASCOT engine (Matrix Science, London, UK; version 2.2) embedded into Proteome Discoverer 1.4 (Thermo Electron, San Jose, CA) against uniprot human database (include 147897 sequences, download at 20150902). For protein identification, the Mascot search parameters were set as follows: Peptide mass tolerance: 20 ppm; MS/MS tolerance: 0.1 Da; Enzyme: Trypsin; Missed cleavage: 2; Fixed modification: Carbamidomethyl (C), iTRAQ8plex(K), iTRAQ8plex(N-term), Variable modification:Oxidation(M), False discovery rate (FDR)≤0.01 [12]. The average of the reporter ion intensities for the two biological replicates was considered for relative quantification. For the selection of differentially expressed proteins (DEPs), the requirements were (i) identification with at least one unique peptide and (ii) the fold changes≥1.5 or ≤0.67 and a *p*-value<0.05 [13].

### Bioinformatic analysis

Differentially expressed proteins (DEPs) were analyzed according to GO terms for biological process, cellular component and molecular function in the database (http://www.geneontology.org/). To assess functional associations between proteins, differentially expressed in FGFR2-overexpressing osteoblasts, the online tool STRING 10 was applied. Pathways enrichment of proteins clusters were performed according to KEGG pathway database.

### Statistical analysis

Data are given as the mean±SEM. GraphPad Prism Software (San Diego, CA, USA) was used for statistical analysis. Significances of difference between groups were determined by a non-paired Student’s t-test.

## Results and discussion

### Construct of human osteoblasts with stable FGFR2 overexpression

**Fig. 1:**
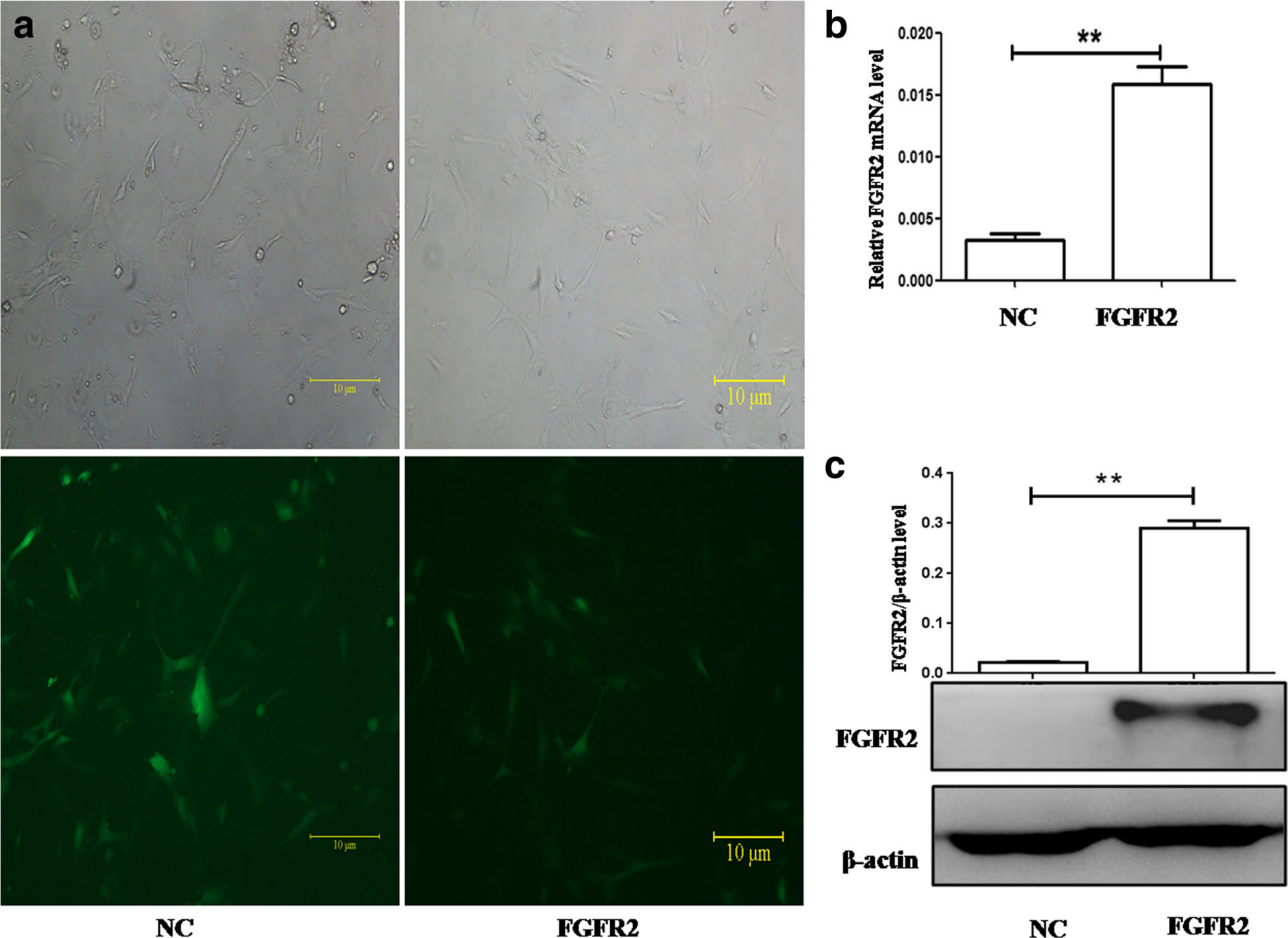
Verification of stable cell lines overexpressing FGFR2 or Control (NC). **(a)**. Phase contrast microscopy (up) and fluorescence images (bottom) showed GFP positive hFOB cells in the same field. Panels shown are: (left) cells transfected with control lentivirus; (b) cells with FGFR2 letivirus. FGFR2 expression was detected after these transfected cells were selected with puromycin. FGFR2 expression was examined by real-time PCR **(b)** and Western blot **(c)**, respectively

To character the proteomic profile regulated by FGFR2, we firstly establish the hFOB11.9 cell line with stable FGFR2 overexpression. The hFOB cells were infected with the lentivirus carrying the humanFGFR2 gene or the empty vector. And the FGFR2-overexpressing (FGFR2) and control (NC) cell sublines were generated by puromycin selection. The efficiency of infection in the two cell sublines was determined by the detection of GFP signals by fluorescence microscopy. As shown in Fig. 1a, green fluorescence was detected throughout the entire cell with high infection efficiency (>95%). Subsequently, the real-time PCR results revealed FGFR2 mRNA level in overexpressing subline was increased 4.84-fold as compared with its control (Fig. 1b). Additionally, immunoblotting result displayed FGFR2 was significantly upregulated in overexpressing subline (Fig. 1c). These results indicated the successful establishment of hFOB11.9 cell lines with FGFR2 overexpression.

### Data analysis and protein identification

In this study, iTRAQ was used to assess proteome changes induced by FGFR2 overexpression. Based on data acquisition, 249, 427 spectra, 14967 unique peptides and 3395 proteins were identified (Additional file 1: Figure S1A). The distribution of peptide numbers ranged from seven to 23 (Additional file 1: Figure S1B). The molecular weights of most proteins were in the range of 10-30 kDa (Additional file 1: Figure S1C). The distributions of peptide length, peptide count, molecular weight and protein sequence coverage were determined (Additional file 1: Table S1).

### Functional annotations of the DEPs

**Fig. 2:**
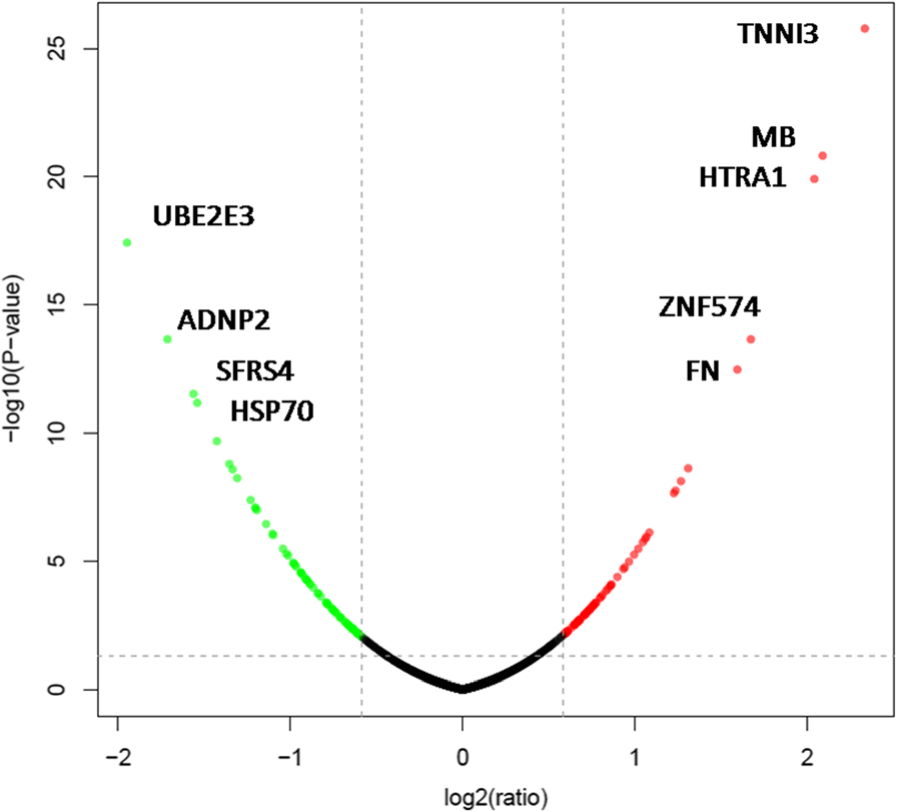
Volcano plot showing the effect of FGFR2 on the differentially expressed proteins in hFOB cells. The vertical dashed line represents the cutoff for fold change and the horizontal dashed line represents the cutoff for the p-value. Proteins upregulated and downregulated with a p-value less than or equal to 0.05 are denoted by green and red colored circles, respectively

**Fig. 3:**
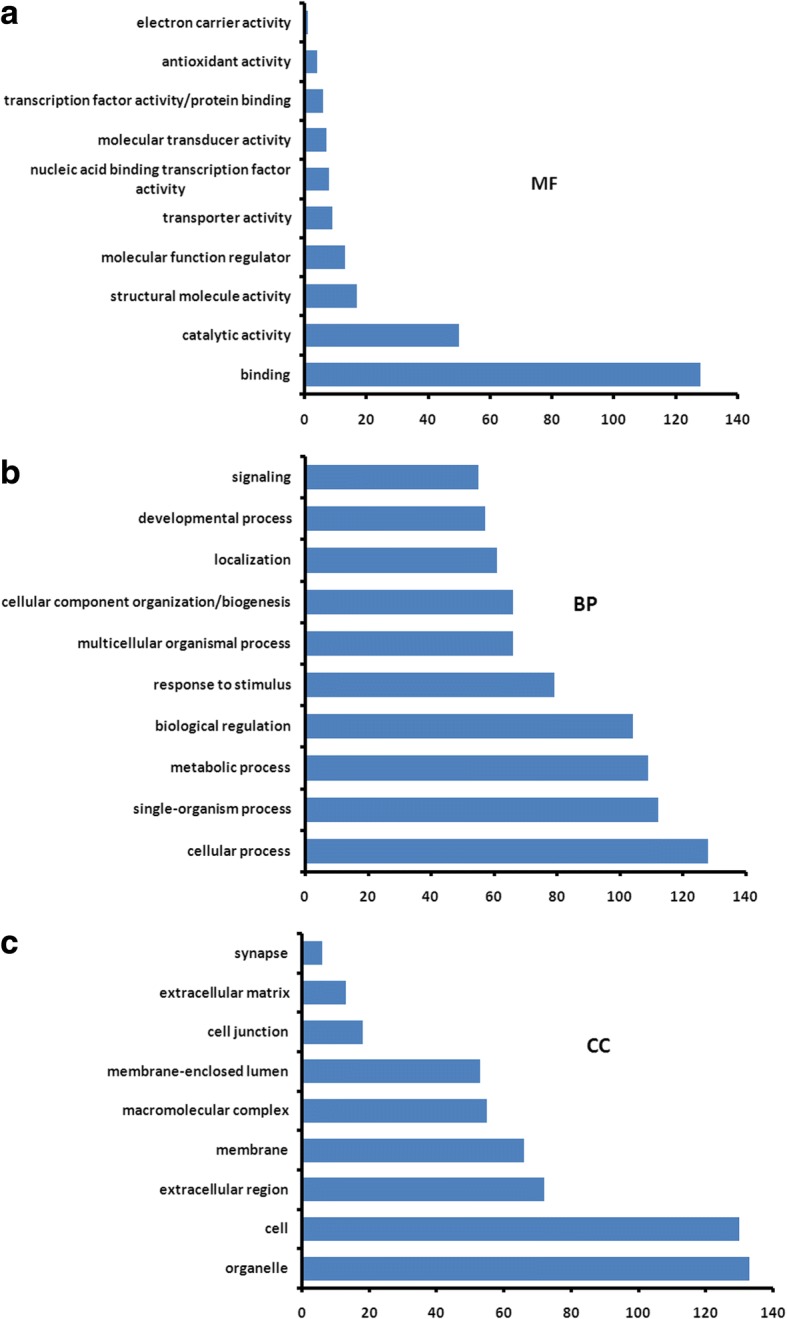
Gene Ontology (GO) analysis of differentially expressed proteins in hFOB cells overexpressing FGFR2. GO analysis was performed according three terms: Molecular Functions (**a**), Biological Process (**b**) and Cellular Component (**c**)

Changes in the protein profile were analyzed and 149 proteins exhibited a difference (fold changes≥1.5 or ≤0.67) with a FDR of less than 0.01% (Fig. 2). Among 149 DEPs, 78 and 71 proteins were significantly up-regulated and down-regulated in the FGFR2-overexpressed hFOB11.9 cells, respectively. GO analysis was performed on these DEPs. For molecular function classification, 10 categories were mainly involved in binding (128 proteins), followed by catalytic activity (50 proteins) and structural molecule activity (17 proteins) (Fig. 3a). Moreover, the binding function mainly included actin monomer binding, protein binding, integrin binding, cell adhesion molecule binding and myosin heavy chain binding (Additional file 1: Table S2). The binding function suggested FGFR2 might regulate osteoblastic biological quality by binding with cytoskeleton or integrin, which in line with previous observation that alpha5 integrin colocalized with FGFR2 and mediated osteoblast detachment and apoptosis [14]. For biological process, the DEPs were related to cellular process (128 proteins), followed by single-organism process (112 proteins), biological regulation (104 proteins) and response to stimulus (79 proteins) (Fig. 3b). Expectedly, differentially expressed proteins were highly associated with extracellular regulation of signal transduction (Additional file 1: Table S3). FGFR2 belongs to tyrosine kinases, which possess three extracellular immunoglobulin-like domains, a trans-membrane region and a cytoplasmic split tyrosine kinase domain, which is activated upon ligand binding. Ligand binding leading to FGFR dimerization, phosphorylation of intrinsic tyrosine residues and activation of several signal transduction pathways [15]. In cellular component category, the DEPs were associated with organelle, cell, extracellular region, cell membrane, membrane-enclosed lumen and macromolecular complex (Fig. 3c). Interestingly, these differentially expressed proteins were mainly located in extracellular region, including extracellular vesicle (exosome) (Additional file 1: Table S4).

### Identification of the DEPs

Among 149 DEPs, 78 and 71 proteins were significantly up-regulated and down-regulated in the FGFR2-overexpressed osteoblasts, respectively. The top 15 up-regulated proteins included ubiquitin-conjugating enzyme E3, ADNP homeobox protein 2 and Homo sapiens heat shock 70kDa protein 6 (Table 1). Of the 15 proteins, UBE2E3 is the most up-regulated protein and its level was increased 3.8-fold in FGFR2-overexpressing osteoblasts. Meanwhile, real time-PCR was performed To further demonstrate DEPs triggered by FGFR2. In line with MS result, the quantitative PCR analysis showed the level of UBE2E3 in FGFR2 group was 3.7-fold higher than that in the control group (Additional file 1: Figure S2), indicating that FGFR2 may influence gene transcription through ubiquitin modification. In another way, the ubiquitin ligase, c-Cbl-mediated negative feedback mechanism controlling FGFR2 degradation in osteoblast differentiation [16]. Thus, the report, together with our result, indicates FGFR2 is involved in ubiquitin modification. Conversely, 71 proteins were significantly down-regulated in the FGFR2-overexpressed osteoblasts and its top 15 down-regulated proteins were listed in Table 2. Among these decreased proteins, several cytoskeleton-related proteins were significantly down-regulated. Cytoskeleton is a cellular scaffolding contained within cytoplasm. It maintains the cell shape, provides mechanical strength, directs locomotion, regulates chromosome separation inmitosis and meiosis and intracellular transport of organelles in cells [17]. In the study, these cytoskeleton-related proteins included Troponin I, Myoglobin, Fibrinogen beta chain, Myosin light chain and Fibrinogen gamma chain. Among these proteins, troponin I (TNNI3) was the most down-regulated protein in FGFR2-overexpressing osteoblasts (5.0 fold, Table 2). In accordance with MS result, the PCR analysis showed TNNI3 expression in FGFR2 group was decreased 3.3-fold as compared with that in the control group (Additional file 1: Figure S2), suggesting FGFR2 regulated Troponin I expression. In agreement with our results, previous study demonstrated FGFR2 regulated Troponin I-inhibited proliferation in endothelial cells [18]. Besides Troponin I, another cytoskeleton-related protein myosin was downregulated in FGFR-mediated myofiber organization [19]. Thus, these observations, together with our study, clearly point to the importance of cytoskeleton in response to FGFR2 in osteoblasts.

**Table 1:** Top 15 increased expressed proteins in FGFR2 overexpressing osteoblasts compared to control

Accession	Description	Gene name	Ratio (FGFR2/NC)	P Value
R4GMM6	Ubiquitin-conjugating enzyme E2 E3	UBE2E3	3.8613	3.67E-18
K7ERT3	ADNP homeobox protein 2 (Fragment)	ADNP2	3.2817	2.22E-14
A8K644	Splicing factor, arginine serine-rich 4, isoform CRA_b	SFRS4	2.9544	3.03E-12
B2R6X5	Homo sapiens heat shock 70kDa protein 6 (HSP70B')	HSP70	2.6893	2.02E-10
O15078	Centrosomal protein of 290 kDa	CEP290	2.5578	1.55E-09
Q8N883	Zinc finger protein 614	ZNF614	2.5237	2.56E-09
A0A024R3R5	Lamin B receptor, isoform CRA	LBR	2.3473	4.06E-08
Q13257	Mitotic spindle assembly checkpoint protein MAD2A	MAD2L1	2.3051	7.94E-08
P02765	Alpha-2-HS-glycoprotein	AHSG	2.301	8.62E-08
E9PKB7	Transcriptional enhancer factor TEF-1	TEAD1	2.2894	1.01E-07
D3DSV0	HCG2043421, isoform CRA_b	hCG_2043421	2.206	3.53E-07
H7BYV1	Interferon-induced transmembrane protein 2	IFITM2	2.1432	9.73E-07
P55081	Microfibrillar-associated	MFAP1	2.0619	3.26E-06
	protein 1			
O14879	Interferon-induced protein with tetratricopeptide repeats 3	IFIT3	2.0328	5.07E-06
A0T1J2	Oxytocin receptor	OXTR	2.019	6.11E-06

**Table 2:** Top 15 decreased expressed proteins in FGFR2 overexpressing osteoblasts compared to control

Accession	Description	Gene name	Ratio (FGFR2/NC)	P Value
A8CN18	Cardiac troponin I	TNNI3	5.0427	1.66E-26
U6FIU7	Myoglobin	MB	4.2561	1.47E-21
Q05DJ8	HTRA1 protein	HTRA1	4.1159	1.20E-20
A0A087WUK5	Zinc finger protein 574	ZNF574	3.189	2.23E-14
P02751	Fibronectin	FN1	3.0201	3.39E-13
D3DP13	Fibrinogen beta chain, isoform CRA_	FGB	2.477	2.33E-09
B4DEY6	LIM and cysteine-rich domains protein 1	LMCD1	2.4059	7.41E-09
O43854	EGF-like repeat and discoidin I-like	EDIL3	2.3544	1.73E-08
	domain-containing protein 3			
P08590	Myosin light chain 3	MYL3	2.3389	2.20E-08
C9JEU5	Fibrinogen gamma chain	FGG	2.1194	7.62E-07
A0A096LNJ1	Protein LOC102724023	LOC102724023	2.0946	1.12E-06
P38936	Cyclin-dependent kinase inhibitor 1	CDKN1A	2.0834	1.35E-06
F4MHI4	Ubiquitously transcribed tetratricopeptide	UTY	2.0641	1.83E-06
	repeat protein Y-linked transcript			
P81605	Dermcidin	DCD	2.0272	3.28E-05
O94819	Kelch repeat and BTB domain-containing protein 11	KBTBD11	1.9528	0.0000104

### Functional pathway analysis

**Fig. 4:**
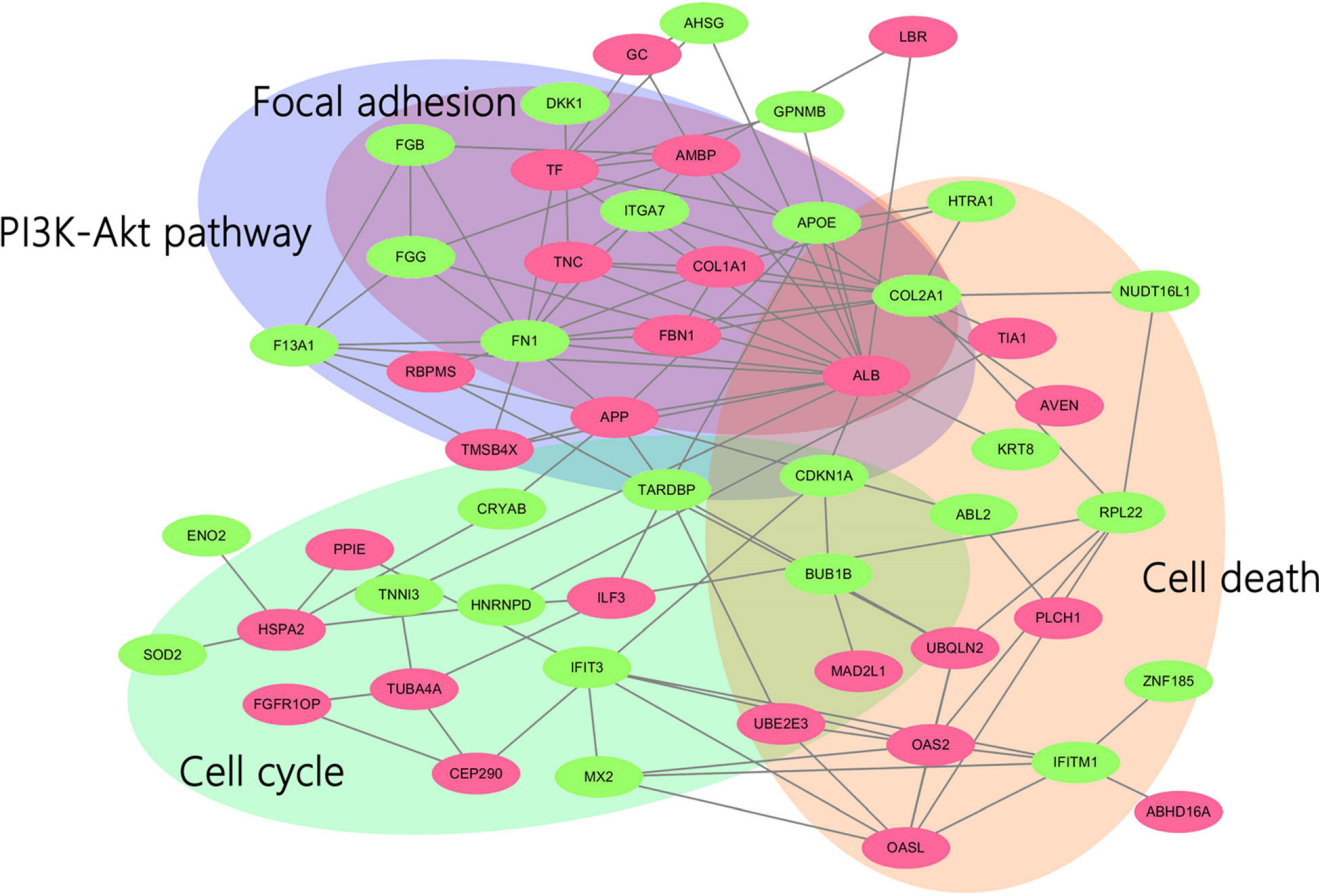
Protein-protein interaction (PPI) network based on DEPs. The round nodes indicate individual proteins. Regulations of protein abundance are shown as red (up-regulation) or green (down-regulation) circles. DEPs involved in specific pathways are organized in big shadowed circles

To obtain functional pathway information, we further analyzed the 149 DEPs using the KEGG database. KEGG pathway analysis identified the signaling pathways of DEPs. These pathways included cell death, PI3K-Akt pathway, focal adhesion and cell cycle (Fig. 4). These networks covered 12 DEPs, including Collagen, type I (ColI), Tenascin (TNC), Cyclin-dependent kinase inhibitor 1 (CDKN1A), Fibronectin (FN1), Centrosomal protein of 290 kDa (CEP290), Heat shock-related 70 kDa protein 2 (HSPA2), Tubulin alpha-4A chain (TUBA4A), Mitotic spindle assembly checkpoint protein (MAD2L1), Mitotic checkpoint serine/threonine-protein kinase BUB1 beta (BUB1B), Interferon-induced protein with tetratricopeptide repeats 3 (IFIT3), Cell death regulator (AVEN) and Abelson tyrosine-protein kinase 2 (ABL2) (Table 3). Of these 12 differentially expressed proteins, 6 proteins (CEP290, HSPA2, MAD2L1, BUB1B, IFIT3, AVEN and ABL2) were significantly increased, some of which play important roles in key processes linked to cell growth and survival [20]. It is thus possible that the effect on osteoblast growth by FGFR2 will result from these upregulated proteins. In addition to the increased expressed proteins, several important proteins, including ColI, TNC, FN1 and CDKN1A, were significantly downregulated. Most of these proteins regulate cell proliferation or survival through focal adhesion and PI3K-Akt pathway. Thus, based on these changed proteins in top-rated networks, FGFR2 overexpression may negatively regulate the osteoblastic biological process, such as cell proliferation.

**Table 3:** KEGG pathway analysis of the identified proteins

Pathway Name	Pathway ID	P value	Genes	Count
ECM-receptor interaction	hsa04512	0.00298	COL2A1;TNC;FN1	3
Protein digestion and absorption	hsa04974	0.00388	SLC1A5;COL2A1;CPB2	3
Cell cycle	hsa04110	0.00946	MAD2L1;BUB1B;CDKN1A	3
PI3K-Akt signaling pathway	hsa04151	0.0314	COL2A1;TNC;CDKN1A;FN1	4
Epstein-Barr virus infection	hsa05169	0.0339	HSPA2;AKAP8L;CDKN1A	3
Focal adhesion	hsa04510	0.0344	COL2A1;TNC;FN1	3
ErbB signaling pathway	hsa04012	0.0383	ABL2;CDKN1A	2

## Conclusion

The iTRAQ technique is a powerful tool for identification of protein isoforms and for comparative proteome studies. In the present study, we identified 149 DEPs triggered by FGFR2 in osteoblasts. These DEPs could be involved in biological processes that lead to cell proliferation or apoptosis. Further studies are necessary to understand the functions of the identified proteins regulated by FGFR2 in osteoblasts. A better understanding of the mechanisms underlying the dysregulation of these proteins may be important for therapeutic purposes in bone related diseases.

## Additional files

Additional file 1: **Figure S1.** Overview of iTRAQ data analysis. (A) The basic statistics of the iTRAQ data. (B) Peptide length distribution. X-axis showed the peptide length, while Y-axis showed the corresponding peptide count. (C) Molecular weight distribution of the identified proteins. X-axis showed molecular weight (kDa), while y-axis showed number of proteins. **Table S1.** Protein list identified in biological replicate 1 and 2 with an FDR of 0.01% at the peptide level. **Table S2.** The categories enriched molecular function (MF) by GO annotation. **Table S3.** The categories enriched biological process (BP) by GO annotation. **Table S4.** The categories enriched cellular component (CC) by GO annotation. **Figure S2.** Real time PCR analyses of TNNI3 and UBE2E3 in hFOB cells overexpressing FGFR2 and control cells. Statistics analysis were performed using non-paired Student’s t-test, with *** representing *p* < 0.01. (ZIP 606 kb)

## Abbreviations

ADNP2: ADNP homeobox protein 2
CDKN1A: Cyclin-dependent kinase inhibitor 1
ColI: Collagen I
FGFR2: Fibroblast growth factor receptor 2
FN1: Fibronectin 1
GO: Gene ontology
HSP70: Homo sapiens heat shock 70kDa protein 6
iTRAQ: Isobaric tags for relative and absolute quantitation
KEGG: Kyoto Encyclopedia of Genes and Genomes
LC-MS: liquid chromatography-tandem mass
PI3K: Phosphoinositide 3 kinase
PVDF: Polyvinylidene fluoride
SDS-PAGE: Sodium dodecyl sulfate polyacrylamide gel electrophoresis
TNC: Cardiac troponin I
UBE2E3: Ubiquitin-conjugating enzyme E2 E3
