# Supplementary figures and images for: iTRAQ-Based Proteomic Analysis reveals possible target-related proteins and signal networks in human osteoblasts overexpressing FGFR2
Source: Proteome Sci. 2018 Jun 19;16:12. doi: 10.1186/s12953-018-0140-x (PMC6011184; doi:10.1186/s12953-018-0140-x)

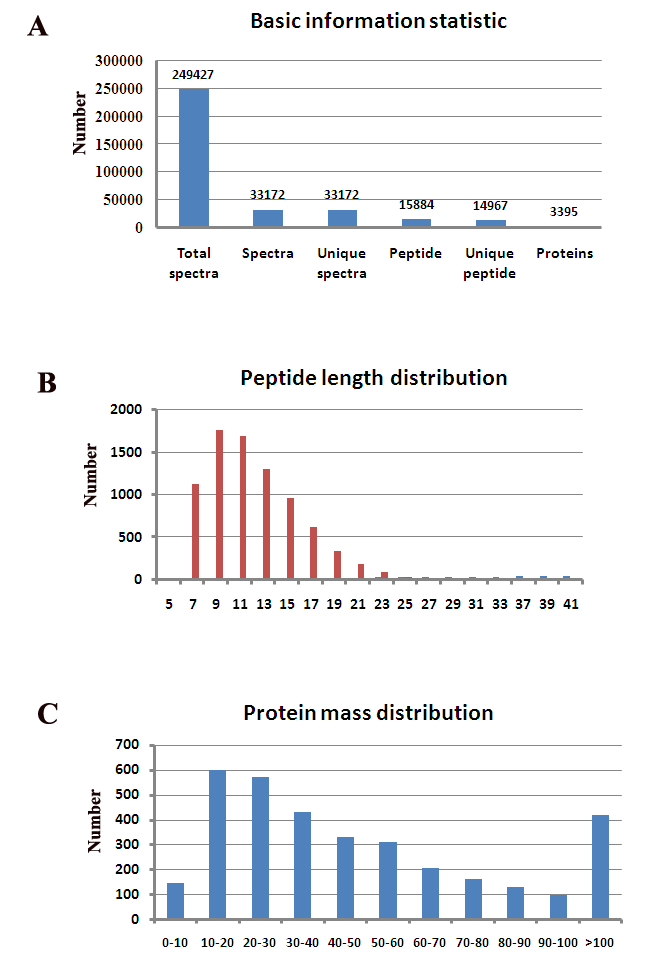

Supplement: Supplementary file 1 — Figure S1. Overview of iTRAQ data analysis. (A) The basic statistics of the iTRAQ data. (B) Peptide length distribution. X-axis showed the peptide length, while Y-axis showed the corresponding peptide count. (C) Molecular weight distribution of the identified proteins. X-axis showed molecular weight (kDa), while y-axis showed number of proteins. Table S1. Protein list identified in biological replicate 1 and 2 with an FDR of 0.01% at the peptide level. Table S2. The categories enriched molecular function (MF) by GO annotation. Table S3. The categories enriched biological process (BP) by GO annotation. Table S4. The categories enriched cellular component (CC) by GO annotation. Figure S2. Real time PCR analyses of TNNI3 and UBE2E3 in hFOB cells overexpressing FGFR2 and control cells. Statistics analysis were performed using non-paired Student’s t-test, with *** representing p < 0.01. (ZIP 606 kb) [file 12953_2018_140_MOESM1_ESM.zip › supplementary Fig. S1.tif]

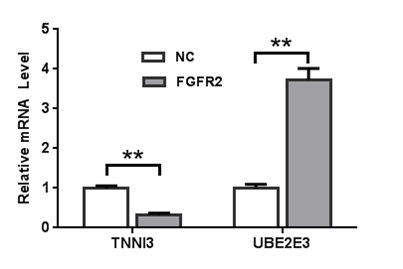

Supplement: Supplementary file 1 — Figure S1. Overview of iTRAQ data analysis. (A) The basic statistics of the iTRAQ data. (B) Peptide length distribution. X-axis showed the peptide length, while Y-axis showed the corresponding peptide count. (C) Molecular weight distribution of the identified proteins. X-axis showed molecular weight (kDa), while y-axis showed number of proteins. Table S1. Protein list identified in biological replicate 1 and 2 with an FDR of 0.01% at the peptide level. Table S2. The categories enriched molecular function (MF) by GO annotation. Table S3. The categories enriched biological process (BP) by GO annotation. Table S4. The categories enriched cellular component (CC) by GO annotation. Figure S2. Real time PCR analyses of TNNI3 and UBE2E3 in hFOB cells overexpressing FGFR2 and control cells. Statistics analysis were performed using non-paired Student’s t-test, with *** representing p < 0.01. (ZIP 606 kb) [file 12953_2018_140_MOESM1_ESM.zip › supplementary Fig.S2.tif]
